# Cross-Sectional analysis of levels and patterns of objectively measured sedentary time in adolescent females

**DOI:** 10.1186/1479-5868-8-120

**Published:** 2011-10-28

**Authors:** Deirdre M Harrington, Kieran P Dowd, Alan K Bourke, Alan E Donnelly

**Affiliations:** 1 Pennington Biomedical Research Center, 6400 Perkins Road, Baton Rouge, Louisiana 70808, USA; 2 Department of Physical Education and Sport Sciences, University of Limerick, Limerick, Ireland; 3 Department of Electronic and Computer Engineering, University of Limerick, Limerick, Ireland

**Keywords:** Sedentary, adolescent females, ActivPAL, sitting, school, methodology

## Abstract

**Background:**

Adolescent females have been highlighted as a particularly sedentary population and the possible negative effects of a sedentary lifestyle are being uncovered. However, much of the past sedentary research is based on self-report or uses indirect methods to quantity sedentary time. Total time spent sedentary and the possible intricate sedentary patterns of adolescent females have not been described using objective and direct measure of body inclination. The objectives of this article are to examine the sedentary levels and patterns of a group of adolescent females using the ActivPAL™ and to highlight possible differences in sedentary levels and patterns across the week and within the school day. A full methodological description of how the data was analyzed is also presented.

**Methods:**

One hundred and eleven adolescent females, age 15-18 yrs, were recruited from urban and rural areas in the Republic of Ireland. Participants wore an ActivPAL physical activity monitor for a 7.5 day period. The ActivPAL directly reports total time spent sitting/lying every 15 seconds and accumulation (frequency and duration) of sedentary activity was examined using a customized MATLAB^® ^computer software programme.

**Results:**

While no significant difference was found in the total time spent sitting/lying over the full 24 hour day between weekday and weekend day (18.8 vs. 18.9 hours; p = .911), significantly more sedentary bouts of 1 to 5 minutes and 21 to 40 minutes in duration were accumulated on weekdays compared to weekend days (p < .001). The mean length of each sedentary bout was also longer (9.8 vs. 8.8 minutes; p < .001). When school hours (9 am-3 pm) and after school hours (4 pm-10 pm) were compared, there was no difference in total time spent sedentary (3.9 hours; p = .796) but the pattern of accumulation of the sedentary time differed. There were a greater number of bouts of > 20 minutes duration during school hours than after school hours (4.7 vs. 3.5 bouts; p < .001) while after school time consisted of shorter bouts < 20 minutes.

**Conclusions:**

School is highlighted as a particularly sedentary setting for adolescent females. Interventions to decrease sedentary time at school and the use of wearable devices which distinguish posture should be encouraged when examining sedentary patterns and behaviors in this population.

## Background

Unlike physical activity, the study of prolonged sitting is a relatively new area of research that has gained significant momentum over the last number of years [1-3]. Currently, the majority of the published literature has utilised screen time [4,5], self-report diaries [6,7] or accelerometer thresholding [8-12] to investigate sedentary lifestyles but the accuracy of these results remain uncertain due to the inherent limitations of each methodology. Screen time represents only one sedentary activity from one domain (leisure time) while self-report methods may underestimate sitting time due to the inability of participants to recall the ubiquitous that is sitting. Furthermore, it is hypothesized that currently employed accelerometer thresholds may overestimate sedentary as it may include standing time [13] but a recent study showed that the commonly used < 100 Actigraph counts threshold actually underestimated sitting time [14].

The intricate relationships between sedentary behaviors, physical activity levels and health have been, and continue to be, uncovered [15-22]. While the relationship between sedentary levels and health have been found to be somewhat independent of physical activity in adults, the relationships between sedentary behaviors and various health parameters is less well established in adolescents [9,10,23-27]. As evidence of the deleterious effects accumulates, undoubtedly intervention strategies will be required to reduce sedentary time [28]. However, little is known about the sedentary levels and patterns of adolescents and how they may accrue their sedentary volume on individual days and over the full week.

Body-worn devices that determine inclination or incorporate activity classification systems and allow for the direct and objective examination of sedentary time are now available. One such device is the ActivPAL™ Professional physical activity monitor (PAL Technologies Ltd, Glasgow, UK) which incorporates Intelligent Activity Classification™ based on information from a micro-machined electrical mechanical system (MEMS) accelerometer. Recorded epochs are classified into periods of sitting/lying, standing and stepping, allowing both sedentary and physical activity to be estimated. The device is gaining credence in the measurement of locomotion and sedentary behavior and is being subjected to extensive validation in a number of population groups [14,29-35]. As technology now exists to allow the direct measurement of body inclination and posture, researchers have emphasised the importance of valid, reliable and objective quantification of sedentary levels and patterns [2,36,37]. In particular, the use of the ActivPAL for measuring sedentary levels has been suggested and recommended as it gives a direct estimate of body inclination (i.e. it does not rely on thresholds or cut-points), allows time spent standing to be estimated independently and can detect changes in sitting/lying time [1,11,14].

The objective of this article is to examine the sedentary levels and patterns of a group of adolescent females using the ActivPAL and to highlight possible differences in these levels and patterns across the week and within the school day. A full methodological description of how the data was analyzed is also presented.

## Methods

The aim of this cross-sectional analysis was to randomly choose urban and rural high schools from the mid-west of Ireland and, in turn, randomly choose participants from within each school. A list of all schools in the area were downloaded from the Department of Education (Ireland) website and a random 12 schools were chosen. Six urban schools and six rural schools agreed to participate. If a school declined to participate the next school on the list was approached. Participants were randomly selected from a list of all 15-18 year olds enrolled in each school. Approval was obtained from the University of Limerick research ethics committee. Only individuals who provided full written parental consent and written participant consent could participate in this study. Recruitment and data collection took place in participants' schools in Spring 2009. Height was measured to the nearest 0.25 cm using a portable stadiometer (Seca model 214, Seca Ltd., Birmingham, UK) and weight was measured to the nearest 0.1 kg using a portable electronic scale (Seca model 770, Seca Ltd., Birmingham, UK) following standard procedures. Body mass index was calculated as weight (kg)/height (m)^2^. Each participant was then provided with an ActivPAL and given a full verbal and written demonstration of its use. The device was only to be removed for bathing, swimming or any other water-based activity. ActivPAL data were collected 24 hours a day for 7.5 days with the first day and last half day of the accelerometry protocol excluded. The accelerometers were retrieved from participants on the 8^th ^day at their schools.

### Wearable Accelerometer Based Device

**Figure 1:**
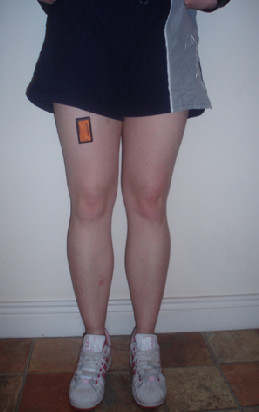
**ActivPAL placement**.

The ActivPAL is a single unit measuring 53 × 35 × 7 mm and weighing 20 grams and incorporates a uniaxial accelerometer. The device measures bodily accelerations and, using the inclination on the thigh, identifies the posture which the wearer is in. The information is allocated into epochs of sitting/lying, standing and stepping using the on-board microprocessor. The device samples at 10 Hz and data are pre-processed, compressed and recorded. It is worn on the midpoint of the anterior aspect of the right thigh and is attached to the skin directly using a PAL*stickie*; a hydro-gel adhesive pad (Figure 1). The ActivPAL has previously been validated for the determination of static, dynamic and postural activity in adults [14,33,34] and adolescent and adult females [29].

### Data Processing

According to prescribed accelerometer testing protocol, participants were required to provide at least 4 days of valid data (including one weekend day) for their data to be included in this free-living analysis [38]. A valid day was considered to be 600 minutes or more of recording during daytime hours (i.e. 7 am to 10 pm). No method to distinguish non-wear time from wear time for the ActivPAL existed. While extended periods of non-wear time rarely occurred in this group of adolescent females, it was felt that an appropriate method, similar to what is available for the Actigraph, was needed. As time spent sitting/lying can produce counts (for example when fidgeting or when moving lightly while sleeping) it was decided that extended periods of continuous zero counts produced while in the posture of sitting/lying would reflect non-wear time. Therefore, non-wear time was defined as 60 minutes or more of continuous unbroken 0 counts from the output data file. When this time was identified, it was omitted and the 24-hour day adjusted accordingly. Total hours spent sitting/lying and standing over the full 24-hour monitoring period was summed directly from the ActivPAL daily and weekly output for each participant. Sedentary levels were also expressed as the total and percent amount of time spent sedentary during the full 24 hours (which included time spent lying while sleeping).

A customised MATLAB^® ^(version 7.0.1, Mathworks Inc, Natick, MA, USA) computer software programme was developed to process the ActivPAL data output files. This program provided detailed information on sedentary patterns accumulated throughout the measured day. ***Number and Length of Sedentary Bouts: ***The programme examined each epoch which contained a full 15 seconds of sitting/lying, and classified this as the beginning of a sedentary bout. This sedentary bout continued until the next 15 second epoch of standing or stepping was identified; for example, when a participant was sitting/lying and then moved into standing or stepping for 15 seconds and longer. The number of sedentary bouts and mean length of the sedentary bouts per day were then calculated. ***Breaks in Sedentary Activity: ***Breaks in sedentary activity represented the times when the participant was not sedentary (i.e. time spent standing or stepping that lasted at least 15 seconds). The number of breaks in sedentary activity and the mean length of each break in sedentary activity were calculated. Although standing expends < 1.5 METs, and therefore could be classed as a sedentary behavior, it was considered to be a break in sedentary activity for the purposes of this analysis. Standing has previously been considered a break in sedentary behavior in adults [39] and so may have its own protective effect. During standing, the contractions of the large muscle groups (quadriceps and hamstrings) promote lipoprotein metabolism [3] and standing increases energy expenditure above resting rates, as people rarely stand perfectly still (people fidget or perform upper body movements).

### Statistical Analysis

Descriptive statistics (means, standard deviations, standard errors (SE) and ranges) were calculated for all variables. Tests for normal distribution were completed on all variables and non-normal variables were log_10 _transformed, providing distributions that more closely approximated normal. Mixed model ANOVA's were used to investigate whether differences existed in the sedentary variables between weekdays and weekend days and between school time and after school time. All analyses were controlled for school, urban vs. rural and number of valid days. Where necessary, analysis was run using both log transformed and untransformed variables. The data from the untransformed variables are reported for ease of interpretation, as the ANOVA results were not notably different. All statistical analyses were completed using PASW Version 18.0 for windows (SPSS Inc, Chicago, IL, USA).

## Results

### Participants

Initially, 140 adolescent females were invited to participate and overall, 111 participated in the study. Reasons for non-participation were being absent either before or after giving consent (n = 21), declining to participate after consenting (n = 6) and 2 adolescent females had sporting commitments on the day of testing. From this sample of 111 participants, 8.1% (n = 9) did not contribute at least 4 days of valid recorded accelerometer data which included at least one weekend day and so were excluded. Of the remaining 102 participants, 24 contributed 4 days, 61 contributed 5 days and 17 contributed 6 days of valid accelerometer data which included at least one weekday. Six days was the maximum number of recorded days that any participant could have. Participant characteristics for the remaining 102 adolescent females are shown in Table 1.

**Table 1:** Participant characteristics of 102 adolescent female participants (means, standard deviations and ranges)

	Mean (± SD)	Range
Age	15 y 11 mo (11 mo)	15-18 y
Height (m)	1.60 (0.10)	1.51-1.81
Weight (kg)	58.2 (9.9)	42.4-89.8
Body mass index (kg/m^2^)	21.7 (3.1)	16-30.0

### Sedentary Levels

Table 2 describes participants' sedentary levels on weekdays and weekend days. No significant differences were identified in total time spent sitting/lying (p = .911) or the percentage of the 24-hour day (p = .913) between weekdays and weekend days.

**Table 2:** Descriptive statistics for sedentary variables compared between weekday and weekend day (mixed model single factor ANOVA) of the 102 adolescent female participants.

	Weekday Mean (± SE)	Weekend Day Mean (± SE)	P =
Total Sitting/Lying (hrs)	18.8 (0.1)	18.9 (0.2)	.911
% of Total day Sedentary	78.4 (0.5)	78.5 (0.7)	.913
Number of Sedentary Bouts (per day)	53 (1)	49 (1)	< .001
Mean Length of Sedentary Bouts (mins)	9.8 (0.2)	8.8 (0.2)	< .001
Number of Breaks (per day)	55 (1)	50 (1)	< .001
Mean Length of Break (mins)	6.5 (0.2)	6.7 (0.3)	.318

SE-Standard Error; All analysis controlled for school, urban/rural and number of valid days

### Patterns of Sedentary Behavior

Table 2 also describes the manner in which sedentary time is accumulated. Significant differences existed in the number of sedentary bouts accumulated, with participants accumulating more sedentary bouts on weekdays compared to weekend days (53 vs. 49 bouts; p < .001). Significantly less sedentary breaks (i.e. when participants transition from sitting/lying to standing and stepping) occurred on weekend days compared to weekdays (55 vs. 50; p < .001) and this reflects the lesser number of sedentary bouts accumulated on weekend days. While no difference was found in the mean length of the breaks in sedentary behaviour (p = .318), significant differences were observed in the mean length of sedentary bouts between weekdays and weekend days (9.8 vs. 8.8 minutes; p < .001).

**Figure 2:**
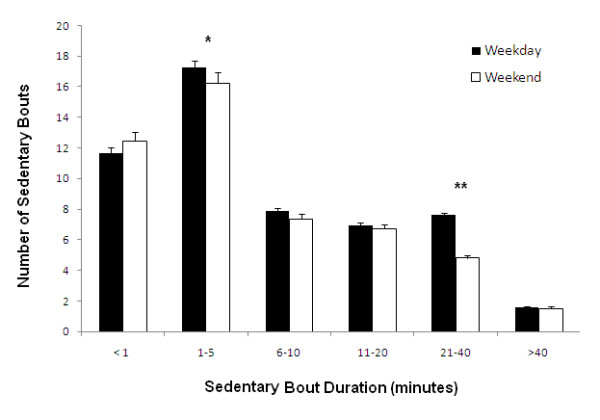
**Number of sedentary bouts (error bars represent SE) on weekdays and weekend days categorized into sedentary bouts of various duration (x-axis)**. Significant difference * (p < .05), ** (p < .001) between weekdays and weekend days.

The mean length of the sedentary bouts masks the fact that some sedentary bouts are extremely short, while others are much longer. Aggregated sedentary bout data were split into separate days for each participant and data were then allocated into bouts of various durations ranging from less than one minute to > 41 minutes (Figure 2). There were significantly less (p < .001) sedentary bouts of 21 to 40 minutes in duration on weekend days (4.8 ± 0.2) compared to weekdays (7.6 ± 0.1) and there were also significantly less sedentary bouts lasting 1 to 5 minutes in duration (p = .03) on weekend days (16.2 ± 0.7) compared to weekdays (17.3 ± 0.4). No differences were found in the bouts lasting < 1, 6 to 10, 11 to 20 or > 40 minutes (p > .05).

**Figure 3:**
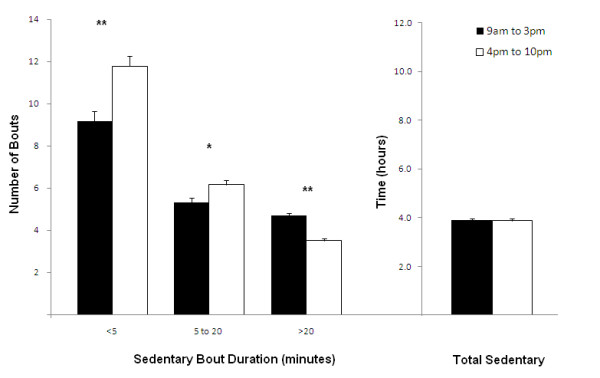
**Number of sedentary bouts (error bars represent SE) on weekdays categorized into sedentary bouts of various duration (x-axis) comparing school hours (9 am-3 pm) to evening hours (4 pm-10 pm)**. **Total time spent sedentary during waking hours is also compared between the two time periods.** Significant difference * (p < .01) ** (p < .001) between 9 am-3 pm and 4 pm-10 pm.

To establish whether certain periods of the day showed differences in sedentary patterns, each day was further broken down from 9 am-3 pm and 4 pm-10 pm representing school hours and after-school hours respectively (Figure 3). These specific periods were examined to compare sedentary levels and patterns when participants had little volition (during school hours) with when they had more free-time (in the evenings at home). As the hour immediately before and immediately after school would have included commuting time, they were excluded from this section of the analysis only. When these time periods were compared, there was no difference in the total number of hours spent sedentary between time periods (3.9 hours; p = .796). However, significantly greater number of longer bouts (> 20 minutes) were accumulated during school hours (4.7 vs. 3.5 bouts; p < .001) and a greater number of shorter bouts of 5 minutes and less (9.2 vs. 11.8 bouts; p < .001) and 5 to 20 minutes (5.3 vs. 6.2 bouts; p = .002) were accumulated in the evening after school.

## Discussion

Due to the recent and increasing evidence of the adverse effects of sedentary behaviors in adults, accurate and valid measures of the full range of sedentary behaviors are clearly beneficial for all populations [37]. This research has directly examined sedentary levels of adolescent females and has described the patterns of accumulation over 24-hours on weekdays and weekend days and across the school day. For the first time, an accelerometer based inclinometer, which has been recommended [1,11], was used to describe these sedentary patterns. A description of the methodology used to analyze ActivPAL output was also presented in the hopes of illuminating the intricate patterns of an activity as ubiquitous as sedentary behavior. Patterns have been looked at in greater detail, in 5 minute blocks, as recommended by past studies which have simply broken down the day by hour [40]. The use of these methodologies can easily be employed when examining sedentary behaviors in all populations, in the hopes of closing the gaps in objectively describing the sedentary profiles of free-living populations.

Analysis from a representative sample of the United States population [11] and a cross-section of 9 European countries [41] highlighted older adolescents as a particularly sedentary group. Similarly, results reported in other objective examinations of sedentary behavior in adolescent populations found the majority of the waking day and the full 24-hour day were spent sedentary [8,42-44]. The results of the present study are consistent with these findings. Aside from television viewing habits, little is known about the pattern of sedentary behavior in adolescents across weekdays and weekend days. Jago *et al *found that adolescent females spent differing number of minutes per hour sedentary throughout weekdays and weekend days with no definite trend obvious [8]. As adolescents engage in high levels of volitional sedentary pastimes [7] one may assume adolescents to be more sedentary in their own leisure time, at evenings and on weekends (when they had the freedom to choose these sedentary pastimes). Participants spent similar amounts of time sedentary on both weekdays and weekends, unlike results from previous research on younger adolescent females [43] and children [40]. However, the present data indicated that these adolescent females were sedentary in longer more continuous bouts on weekdays compared to weekend days. No evidence exists yet to say that these longer bouts have a deleterious effect on young people's health. Sedentary behaviours in general can have an adverse effect on current body composition [45] and may track into adulthood [46] which in turn can increase the risk of coronary heart disease and other co-morbidities [16], and intuitively should be discouraged.

Our findings also highlight the school day (9 am-3 pm) as a particularly sedentary time for this population. To our knowledge, this is the first study to objectively report the manner in which adolescents accumulate their sedentary time while at school using a direct measure of posture. Steele *et al*. reported that 8-10 year old children accumulate 242 minutes of sedentary time during school based hours and this was not different to out of school sedentary time [40]. Regardless of any differences between school and after school, unwittingly, the school setting appears to promote unbroken continuous periods of sitting; periods which have potential deleterious effects on health in adulthood [39]. Our results indicate that while there was no difference in the volume of sedentary time, these adolescent females broke up their sedentary periods more outside of school rather than during school which is encouraging. The duration of each classroom based lesson in these schools ranged from 25 to 40 minutes, resulting in students sitting, uninterrupted, for these lengths over many classes for the majority of each day. School-based interventions which have the primary goal of decreasing sitting time and which have a sedentary message that is independent of other health behavior messages (e.g. physical activity) are lacking [47] and warranted [36] in the literature. We have identified times of day when adolescent females are particularly sedentary and have given a magnitude to the amount of sitting/lying that is done which can inform interventional design. While it may not always be feasible to reduce total sedentary time during school, identifying these typical or 'usual' unbroken bouts offer an easier or more acceptable approach for school-based sedentary interventions.

Inclinometer based measures of sedentary time, such as the ActivPAL, provides researchers with a real time, accurate and objective measure of sedentary behavior, providing actual inclination information as it occurs. More researchers are turning to these direct and objective methods due to the lowering costs of these devices, coupled with the rich and descriptive output obtained, such as the data that has been presented herein. The utility of methodologies using the ActivPAL can be best evaluated and understood when they are compared with the existing published methods of examining sedentary levels and patterns which have already been mentioned in the Background. In highlighting the limitations of past studies, the limitations of the present study also need to be considered. Although this was a random, cross-sectional sample, the size of the sample suggests the results must be interpreted with caution. The results are also descriptive in nature and may not be extended to other populations or age groups at this stage. We have presented total sitting/lying time over the full 24-hour day, but without a self-report log for participants to report at what time they got up and went to bed, we cannot make any conclusions on total time spent sitting during waking hours to compare with past studies. This value for 24-hour sedentary time will be higher than previous studies not only because it includes time spent sedentary after a participant goes to bed but also includes time spent lying (not just sitting time) during waking hours. While this does make comparisons with past accelerometer results difficult, it gives a total value for sedentary, including time spent lying down during the day and night, for this group of adolescent females. Also, the absence of accurate health markers does not allow conclusions on the relationship with sedentary patterns and behavior to be drawn. However, these results do give a more detailed insight into the patterns of sedentary behavior of adolescent females. The limitations of the sample should not detract from the presentation of rich, detailed information that can be garnered from an objective device such as the ActivPAL. Continuous data such as this, combined with robust physiological and health measures, could begin to identify whether a health-compromising level of sedentary time exists.

## Conclusions

This study is one of the first to have used the ActivPAL to objectively quantify sedentary levels and patterns without the need for thresholds, in a group of adolescent females. This article identified that adolescent females accumulate a greater number of sedentary bouts, and of longer duration, on weekdays compared to weekend days. The article also highlighted that while there is no difference in the volume of sedentary activity participants engage in between school hours and after school hours, the pattern of accumulation is different. For future use, a full methodological description of how the data was analyzed was presented. The results emphasise the importance of the development of school based interventions to decrease sedentary bout duration during school hours.

## Competing interests

The authors declare that they have no competing interests.

## Authors' contributions

DH and AD developed the study concept. DH and KD designed the study, collected the data, and drafted the manuscript. DH, KD and AB analysed the data. All authors ran the statistical analysis and read and approved the final manuscript.
